# Reduction of Natural Killer but Not Effector CD8 T Lymphoyctes in Three Consecutive Cases of Severe/Lethal H1N1/09 Influenza A Virus Infection

**DOI:** 10.1371/journal.pone.0010675

**Published:** 2010-05-18

**Authors:** Laura Denney, Celia Aitken, Chris Ka-Fai Li, Eleri Wilson-Davies, Wai Ling Kok, Colin Clelland, Kevin Rooney, Duncan Young, Tao Dong, Andrew J. McMichael, William F. Carman, Ling-Pei Ho

**Affiliations:** 1 Medical Research Council Human Immunology Unit, Weatherall Institute of Molecular Medicine, Oxford University, Oxford, United Kingdom; 2 West of Scotland Specialist Virology Centre, Gartnavel General Hospital, Glasgow, United Kingdom; 3 Pathology Department, John Radcliffe Hospital, Oxford, United Kingdom; 4 Adult Intensive Care Unit, John Radcliffe Hospital, Oxford, United Kingdom; 5 Intensive Care Unit, Royal Alexandra Hospital, Paisley, United Kingdom; 6 Oxford Centre for Respiratory Medicine, Oxford Radcliffe NHS Trust, Oxford, United Kingdom; Karolinska Institutet, Sweden

## Abstract

**Background:**

The cause of severe disease in some patients infected with pandemic influenza A virus is unclear.

**Methodology/Principal Findings:**

We present the cellular immunology profile in the blood, and detailed clinical (and post-mortem) findings of three patients with rapidly progressive infection, including a pregnant patient who died. The striking finding is of reduction in natural killer (NK) cells but preservation of activated effector CD8 T lymphocytes; with viraemia in the patient who had no NK cells. Comparison with control groups suggests that the reduction of NK cells is unique to these severely ill patients.

**Conclusion/Significance:**

Our report shows markedly reduced NK cells in the three patients that we sampled and raises the hypothesis that NK may have a more significant role than T lymphocytes in controlling viral burden when the host is confronted with a new influenza A virus subtype.

## Introduction

The recent H1N1/09 swine-origin influenza pandemic appears to have a high infectivity rate but low pathogenicity [1]. However, it is clear that some subgroups of patients have a poorer outcome – the Mexico outbreak suggested that younger patients, especially those with co-morbidities and morbidly increased BMI were more susceptible to respiratory failure [2]. More recently, surveillance study from 2005 to April 2009 in that area confirms that peaks for mortality and morbidity in the new H1N1/09 strain, have shifted from the very old and young (below 5 and above 80 years of age) to those between the ages of 5 and 59 years, with a focused peak in both deaths and pneumonia in the 20 to 44 age group [3]. The cause of severe illness in this compared to the older age groups is unclear. There is some evidence to suggest lack of cross-reactive immunity from previous subtypes of influenza A viruses, though neither this nor the actual mechanism of immune protection is clear. Studies in mice suggest that T lymphocytes and the innate immune arm (monocytes, macrophage and neutrophils) are involved in both immune-protection and pathology [4] but the factors involved in protection in some patients and tissue injury in others and the relative contribution of various immune cell subsets to these outcomes have not been established in mice or humans. The current pandemic and samples stored from patients offers an opportunity to clarify some of these questions and ultimately improve management of severely ill patients with influenza virus infection. To provide one starting platform, we report here the salient clinical features and immunological profile from three cases of severe illness from patients in this peak mortality age group, sourced from the first outbreak in UK, including histology of lung sections from post mortem study of one fatal case.

## Methods

### Case patients

The cases reported here (Patient 1–3) represent the first wave of patients admitted to Intensive Therapy Unit (ITU) in the first UK outbreak of H1N1/09. Clinical data were presented in detail and captured from case notes.

Blood sample for isolation of peripheral blood mononuclear cells (PBMCs) was obtained during the period of acute illness period in Patient 1, and after clinical recovery in Patient 2 and 3 (see clinical course for timings).

### Control groups

As a comparison and control for these flow cytometric findings, PBMCs from seven healthy, uninfected individuals [mean 32.6 (S.D. 8.9) years old]; three healthy, uninfected pregnant women (35, 31 and 34 years old; 37,26 and 35 weeks pregnant respectively) and four patients who had confirmed H1N1/09 infection but did not require mechanical ventilatory support (‘H1N1/09, non-ITU’). Of the four, one was 29 years old with no previous illness and not hospitalised; the other three were 55, 26 and 47 years of age, and hospitalised for 4–10 days. They presented with mild dyspnea, fever and cough; and recovered quickly without requiring ITU or mechanical ventilatory support. One patient also had asthma, the others had no concomitant clinical conditions. All samples were collected within three days of hospital admission (the non-hospitalised subject was also sampled within three days of symptom onset). Three patients admitted to Intensive Therapy Unit and ventilated for other conditions [post-abdominal surgery (anterior resection), pneumonia, and post-esophagectomy] provided a comparison for severe illness requiring ITU support (labelled as ‘non-flu, ITU’). These samples were also analysed using similar conditions; and cells were extracted and stored using the same methods. All individuals in comparison groups were clinically assessed by a senior respiratory physician.

### Cellular immunology profile in peripheral blood

PBMCs were obtained using Ficoll separation and stored in DMSO/FCS at −80°C according to previously described methods [5].

To determine the T lymphocyte profile, we examined the proportion of activated effector CD8 T cells (as described by Miller JD et al [6]) using a combination of CD38, CD8 and HLA DR mAb staining by flow cytometry. Influenza virus-specific CD8 T lymphocytes in the peripheral blood were determined using MHC-1 tetrameric complex (HLA*A0201) loaded with peptide (GILGFVFTL) derived from the conserved M (Matrix M58-66) virus protein, using methods first established in our laboratory [7], [8]. Two of the three infected, ventilated patients, one of the three infected, non-ventilated, and three healthy subjects were HLA*A0201, permitting the use of this reagent on their blood. We also examined the CD4:CD8 T lymphocyte ratio, the proportion of inflammatory monocyte (CD14^+^ and CD16^+^) [9] and neutrophil (FSC/SSC configuration and CD11b), invariant natural killer T cells (CD3^+^, Vβ11^+^ and 6B11^+^) [10], and natural killer cells (CD3^−^ and CD56^dim^ and CD56^bright^) [11]. Dead cells were gated out with Hoechst staining [12]. All antibodies were obtained from eBiosciences (Hatfield, UK). Methods for flow cytometry staining are as previously described [5], [13].

In order to examine if the freeze and thaw procedure disproportionately affected viability of the cells, we compared staining of fresh and stored samples from all of the healthy, uninfected individuals using the same panel of mAbs; and also from five patients with acute seasonal influenza virus infection to examine storage effects on activated immune cells which may be more sensitive to cell death.

### Examination of post mortem lung sample

Post mortem lung sample from Patient 1 was stored in paraformaldehyde and embedded in paraffin for haematoxylin and eosin staining. Identity of the cell infiltrates was determined with a combination of haematoxylin staining and immunohistochemistry staining for CD68 (macrophages), CD3 (T cells), CD56 (NK cells) and CD20 (B cell) after antigen retrieval using microwave and citrate or EDTA buffer methods [14]. All were detected using a two-step peroxidase staining kit (Dako UK) according to manufactory instruction and as described previously [14]. Appropriate controls (including uninfected normal lungs sections) were used for all staining.

### Diagnosis and detection of virus in samples

Serial respiratory and EDTA plasma samples were obtained from all patients admitted to the ICU and first tested using a validated in-house generic influenza A real time assay based on the Matrix gene, and confirmed with swine-origin H1N1/09 specific Matrix gene as recently described [15]. The crossing threshold (Ct) value (at the inflexion spot of the sigmoid amplification curve to capture the point where DNA amplification is exponential) was recorded for all positive results. This represents positive pick-up of RNA against blank (no RNA). Ct for the ‘no RNA’ control was greater than 40. In Patient 1, post mortem lung, lymph nodes, spleen, liver, distal small bowel and kidneys were also examined for presence of swine-origin H1N1/09 viral RNA. These results were compared with 52 paired blood and nasopharyngeal swabs obtained from community cases of H1N1/09 infection during the first phase of the Scottish outbreak in July-August 2009.

### Ethics

Patients 2 and 3, and the next of kin of Patient 1 gave informed written consent for the studies performed on their samples and publication of their cases. Participation of the control cases was approved by the Oxfordshire Research Ethics Committee.

## Results

### Relevant patient clinical details

**Figure 1:**
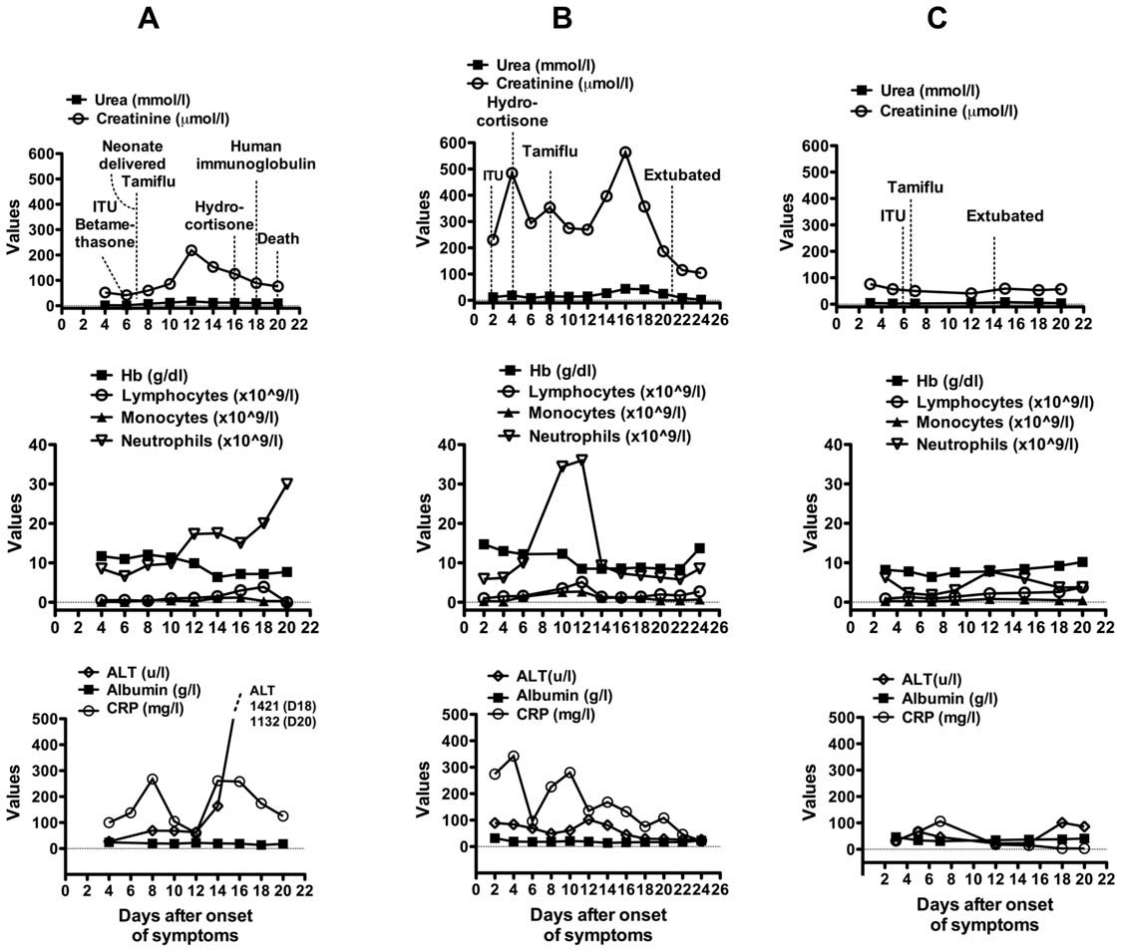
Longitudinal changes in organ function from onset of symptoms (Day 1). Biochemical and haematological indices were captured every two days throughout disease course for Patient 1 (column A), Patient 2 (column B) and Patient 3 (column C). Relevant clinical and therapeutic interventions are shown on the urea and creatinine serial graph for each patient. Normal levels are Urea – 2.5–7.5 mmol/l; Creatinine - 40–139 umol/l; Hb - 12.0–17.5 g/dl; lymphocytes – 1.5–3.5×10^∧^9/l; monocytes 0.2–0.8×10^∧^9/l; neutrophils 2.5–7.5×10^∧^9/l; ALT- 0–50U/l; albumin - 32–45 g/l; CRP - 0–10 mg/l.

All patients had swine-origin H1N1/09 influenza, detected as described above, and received Oseltamivir. Details of longitudinal changes in organ function, haematological indices and inflammatory markers are shown in Figure 1. Admission drugs, radiologist reports of presenting chest radiograph (CXR) and absolute blood leukocyte counts at the point of sampling for PBMCs are displayed in Table 1. All reference to ‘Days’ in text and figures denote days after onset of symptoms. None of the patients were knowingly in contact with infected or exposed individuals or travelled abroad in the few months prior to admissions.

**Table 1:** Admission drugs, Radiologist Reports of Presenting chest radiograph (CXR) and absolute blood leukocyte counts at the point of sampling of PBMC used for phenotyping.

	Patients		
	1	2	3
Age	38	38	24
Gender	F	M	F
Presenting CXR	Ill defined patchy consolidation in right and left lower zones	Patchy bilateral upper, mid and lower zones consolidations	Extensive right lower lobe consolidation and left lower zone patchy consolidations
Lymphocytes (x10^∧^9/l) (normal: 1–4)	3.9	2.6	2.6
Neutrophil count (x10^∧^9/l)(normal: 2.5–7.5)	19.9	7.4	3.0
Monocytes count (x10^∧^9/l) (normal 0.2–0.8)	0.3	0.9	0.4
Drugs on admission to hospital	Aspirin 75 mg Folic acid 5 mg Ferrous sulphate 600 mg	Orlistat 360 mg Simvastatin 40 mg Aspirin 75 mg Omeprazole 20 mg Lisinopril 20 mg Frusemide 40 mg Carvedilol 12.5 mg Nitrazepam 5 mg	None

### Clinical course

#### Patient 1

**Figure 2:**
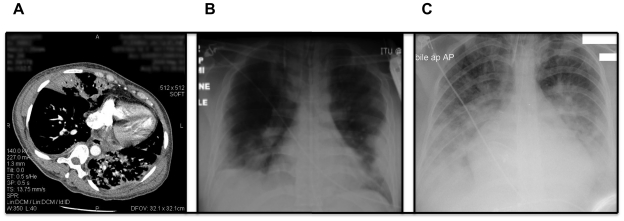
Radiographic images at presentation for Patient 1–3. Cut (mediastinal window) from mid thoracic region of the CT pulmonary angiogram of Patient 1 showing bilateral widespread nodular consolidation (A). The CTPA was reported as showing extensive inflammatory change in both lungs with solid consolidation of both lower lobes and right middle lobe; with widespread patchy infiltration in both upper lobes. CXR for Patient 2 (B) on day of intubation showed bilateral mid and lower zone consolidations, and possible left pleural fluid collection; and for Patient 3 (C) – extensive right lower lobe consolidation and patchy consolidation in left lower lobe; enlarged cardiothoracic ratio and evidence of pulmonary edema.

38 yr old woman, smoker (25–40 cigarettes/day), 29 weeks pregnant; previous history of left parietal lobe infarction in 1999 secondary to high-grade stenosis of left carotid artery, resulting in residual right-sided hemiplegia and minor expressive dysphasia. Presented with three to four days history of progressively worsening dyspnoea, pyrexia and cough. She was admitted on Day 4 and started on broad-spectrum antibiotics. A CT pulmonary angiogram on Day 5 excluded pulmonary embolism but showed extensive bilateral lower lobe pneumonic consolidation (Figure 2a). She was intubated on day 6 after rapidly progressive respiratory failure. Neonate was delivered on Day 7. Progressive deterioration in liver function was recorded from Day 8, and renal impairment from day 10. Blood sample for PBMC was collected on Day 18. Patient died 2 days later from multi-organ (lungs, liver, heart) failure. CXR series showed progressive, widespread consolidation throughout disease course. Post mortem was performed 48 hours later, and lung samples collected. Viral RNA was detected in blood on day 7, 11 and 13 after admission, and in post mortem lungs, blood and lymph nodes but not in any other organs (Table 2). Repeated blood cultures, tracheal aspirates and line tip cultures showed no bacterial growth.

**Table 2:** Viral RNA in Samples from Patients 1–3.

		Respiratory samples(Ct)		Blood samples(Ct)	Post Mortem (Patient 1 only) (Ct)				
	Days in ITU	Naso-pharyngeal swab	Endotracheal aspirate	Plasma	Lung	Lymph node	Spleen	Liver	Kidney
Patient 1	1	27.5							
	3								
	6	24.9							
	7	27	24.9	34.6					
	11		25.9	31.9					
	13			31.2					
	PM				34.1	36.3	neg	neg	neg
Patient 2	1	36.3							
	3		25.9						
	5		29						
	10		35.7						
	12			Neg					
	17	Neg							
Patient 3	1	38.4							
	3		Neg	Neg					
	5			Neg					
	7	35.2							
	10	37.6							

Note. – Neg  =  Negative

#### Patient 2

38 year old man, smoker (5 cigarettes/day) with Type II diabetes mellitus, alcohol-related dilated cardiomyopathy (ejection fraction of 60–65% on admission; diagnosed 2001). Body mass index 29.5, HbA1c in April 2009 was 7.2%; plasma glucose usually poorly controlled. Presented with dyspnoea and admitted directly to ICU on arrival to hospital. CXR on admission showed patchy areas of consolidation bilaterally (Figure 2b) and increased opacification laterally which may be caused by collection of pleural fluid or dense consolidation (thoracic ultra-sound scan was not performed). There was progressive and extensive nodular airspace opacification but some improvement was reported from Day 13, though these changes persisted until Day 22. Broad-spectrum antibiotics were commenced on Day 2. Disease course was complicated by renal and liver function impairment; neither was abnormal before admission (Figure 1b). He was well enough to be extubated and discharged to the ward on Day 22 and went home on Day 27. Blood sample for PBMC was obtained on Day 46. Sputum sample on Day 2 showed heavy growth of Haemophilus influenzae. No other relevant growth in other cultures throughout admission.

#### Patient 3

24 year old woman with underlying mild asthma (no previous hospital admission or regular treatment for asthma), and atrial septal defect repaired in 2007; life-long non-smoker. Admitted to hospital with three days of vomiting, diarrhoea, malaise, fever, headache and dyspnoea. She had productive cough on admission (Day 3) with extensive right lower lobe and patchy left basal consolidation on CXR (Figure 2c). Broad-spectrum antibiotics were started on Day 3. She was transferred to ICU and intubated on Day 6 and remained in ICU for 11 days, with only one organ failure (lungs). Blood sample for PBMC was obtained on Day 34.

### Effect of freeze and thaw on immune cells of healthy and infected samples

We found both CD14^+^ and CD16^+^ monocytes significantly reduced after storage (the latter, thought to represent inflammatory monocytes [9] in humans were more markedly affected). Invariant NKT cells, possibly owing to the rare nature of the cells, showed variable degrees of loss but all other cells remained stable after storage (data not shown). We have therefore not reported on monocytes or invariant NKT cell frequencies.

### Immunological profile in peripheral blood

**Figure 3:**
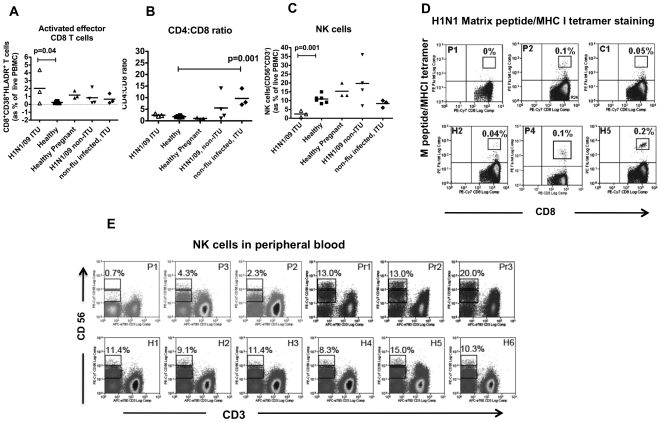
T lymphocyte and NK cell profile in blood of patients. (A -C) Activated effector CD8 T cells, CD4:CD8 T lymphocyte ratio and NK cell frequencies in the three patients (H1N1/09 ITU) compared to the other groups as described in Methods. (D) CD8 T lymphocytes specific to H1N1 matrix epitope as detected by MHC1 tetramer loaded with Matrix peptide. P1 – Patient 1; P2 – Patient 2; C1 – non-HLA A*0201 individual as control (should not stain); H2 and H5 – two healthy individuals with HLA A*0201 genotype; P4 – infected, non-ventilated patient with HLA A*0201 genotype. H5 had symptoms suggestive of influenza virus A infection a few years ago. (E). NK cells identified by CD56^+^ and CD3^-^ staining. NK cells were all CD16^+^ for P2 and 3. P1-3 – Patient 1-3; Pr1-3 – healthy uninfected pregnant women; H1-6 – healthy uninfected individuals. In (D), % refers to frequencies of tetramer^+^ cells as proportion of CD3 T cells. And in (E), both CD56^bright^ and CD56^dim^ subsets as proportion of live PBMCs.

There was an increased proportion of activated effector CD8 T cells in the circulating blood as expected [6], [16] [mean of 2.0±2.2% (S.D.) of live PBMCs compared to 0.25±0.16% in healthy uninfected; p = 0.04, Student's t test], but no expansion of influenza virus specific T cells to Matrix peptide in the peripheral blood, and no significant difference in the CD4:CD8 ratio compared to healthy uninfected population (mean of 2.4±0.5 vs 1.8±0.2; p =  0.06, Student's t test) (Figure 3a,b,d); though the trend was for higher CD4:CD8 ratio in the cases. One healthy volunteer had detectable Matrix peptide-specific T cells (H5); and the one of four H1N1/09 infected, non-ventilated patient who was HLA*A0201 also showed a virus-specific T cell response (P4)(Figure 3d).

Both infected, non-ventilated (n = 4), and the non-infected, ventilated (n = 3) patients showed a markedly raised CD4:CD8 ratio suggesting that increased peripheral CD4:CD8 T cell ratio is a feature of acute illness. This concurs with the fact that 2 of the three infected, ventilated patients (our primary cases) had blood sampled after the acute infection and discharge, while all the other 7 patients were sampled during the acute period of illness, while they were still hospitalised.

Unexpectedly, NK cells were markedly reduced in all three severely ill, ventilated H1N1/09 infected patients (Figure 3c) (mean of 2.4±1.8% of live cells vs 10.9±2.4 in healthy uninfected, p = 0.001; Student's t test); none was found in Patient 1 who was pregnant. Both CD56^bright^ and CD56^dim^ subsets of NK cells were equally reduced. Healthy pregnant women had higher levels of CD56^hi^, which was expected [11] (Figure 3e).

Analysing the NK and CD8 effector T cells in absolute values (ie number of cells/l of blood) rather than proportions, we also found reduced NK cells and elevated CD8 effector T cells – mean (±S.D.) of 72 (30) ×10^∧^6/l vs 153(±14) ×10^∧^6/l for NK cells in cases compared to healthy controls; and 61(±37) ×10^∧^6/l vs 3.6(±0.9) ×10^∧^6/l for CD8 effector T cells in cases compared to healthy controls.

### Post mortem lung findings for Patient 1

**Figure 4:**
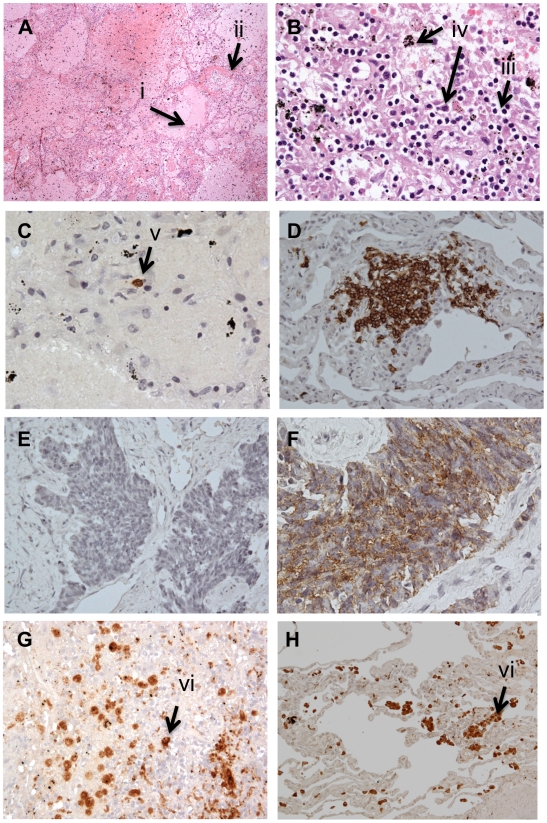
Histopathology sections from lungs of Patient 1. (A) H&E section showing fulminant diffuse alveolar damage (DAD), arrows indicate pulmonary oedema (i) and hyaline membrane formation (ii). (B) High magnification of an area of inflammatory infiltrate showing predominantly lymphocytes (iii) and macrophages (iv). (C)-(H) – Immunohistochemistry staining of lungs showing: (C) occasional CD20 (B cell) staining (v); (D) control showing B cells in a lung section from a patient with rheumatoid nodule (positive cells in dark brown); (E) CD56 (NK cell) staining in Patient 1 showed no positive cells compared to positive control (F) from lung of a patient with non-small cell carcinoma; (G) shows CD68 (macrophage) staining (vi) in Patient 1 compared to lung section from the patient with rheumatoid nodule (H). Negative controls (no primary mAb) for (C)-(J) were all negative. All magnifications are ×100, apart from (A) × 20 and (B) and (C) ×200.

The most striking finding from the haematoxylin and eosin sections is diffuse alveolar damage (DAD) with characteristic pulmonary oedema and fibrin deposition in the alveolar space (Figure 4a). Large areas of haemorrhage were evident throughout a severely injured lung characteristic of fulminant acute respiratory distress syndrome. There were no areas of normal lung parenchyma; inflammatory infiltrates were scarce. Where present, these were predominantly CD3^+^ T cells, with scattered CD20^+^ (B) cells and no CD56^+^ (NK) cells (Figure 3b-d). Macrophages were evident both morphologically and confirmed with CD68^+^ staining (Figure 3e). No infiltrates of polymorphs were observed.

### Viral RNA in patient samples

Viral RNA was detected in nasopharygeal swab and endotracheal aspirate of all patients but only in plasma of Patient 1 (Table 2). None was detected in any of the 52 community blood samples which tested positive for swine-origin H1N1/09 viral RNA on nasopharyngeal swabs. The virus RNA levels remained high in endotracheal secretions for patient 1 throughout the disease course in contrast to Patients 1 and 2; and her plasma viral RNA levels increased with worsening illness.

## Discussion

In this report, we provide a detailed clinical course on three severely ill patients infected with H1N1/09. In the first patient, the progression of disease was quick and her only risk factor was pregnancy. In spite of Betamethasone (administered to enhance maturation of the foetal lungs), 200 mg Hydrocortisone, 20 g human immunoglobulin infusions, high dose Oseltamivir (150 mg bd), Ribavarin and broad-spectrum antibiotics, she succumbed to progressively worsening lung injury and likely disseminated viral infection. She had peripheral lymphopaenia in the early part of her illness (Figure 1a) but there were no abnormalities in the composition of her circulating PBMCs. Most strikingly, H1N1/09 viral RNA was detected not only in the respiratory tract but also in her blood by day 7 of her ICU stay (Day 13 post onset of symptoms on graph, Figure 1a). Increasing level of viral RNA in the blood corresponded with worsening neutrophilia and deterioration in liver function. This could be due to bacterial superinfection at this point and may be contributing factor to the patient's death. For Patient 2, his viral disease was complicated by H influenza pneumonia but despite this, several risk factors, and multi-organ impairment (cardio-respiratory, renal and liver); he made a good recovery. Other than the factor of pregnancy and lack of detection of viral RNA in blood, one major difference was the administration of hydrocortisone early in the course of disease. Patient 1 was administered 12 mg Betamethasone intramuscularly early (on two consecutive days); this dose was equivalent to approximately 64 mg of hydrocortisone (Patient 2 received 200 mg/day for 8 days) (Figure 1a&b). Patient 3 had relatively mild disease compared to Patient 1 and 2 but progressed rapidly to respiratory failure. She had single organ failure and made an uncomplicated recovery. Corticosteroids were not administered.

Like the reports from Mexico [3], all three patients presented with rapidly progressive and widespread pneumonic consolidation on chest X ray. Although it was suggested that the patients in the Mexican outbreak had primary viral pneumonia, it is not possible to confirm this in our three patients as they all received broad-spectrum antibiotics early. However, it is highly likely that this is the case with Patient 1 as there was no evidence of neutrophilic infiltrate in the lung sections.

In the post mortem lungs of Patient 1, the major histopathological finding was diffuse alveolar damage (DAD), reflecting the clinical syndrome of acute respiratory distress syndrome (ARDS). There was complete obliteration of airspace with oedema and haemorrhage but scanty cellular infiltrate – any was composed of T cell and macrophages. Macrophage infiltrate was distinct and calls into question its role in tissue injury. These post mortem changes are very similar to those described for cases from the 1918 pandemic and epidemics since then [17], in the autopsy series on fatal cases of severe acute respiratory syndrome (SARS) [18], and reported fatal H5N1 cases [19].

The blood samples for immunological profile were obtained at different phases of disease [acute in Patient 1 vs recovered (at least 10 days after resolution of symptoms) in Patients 2 and 3]; but in all patients, the NK cells were reduced, in contrast to the effector CD8 T cells which showed no abnormality. In Patient 1, there was a complete loss of NK cells in the peripheral blood. She was also the only patient in whom viral RNA was detected in the blood, raising the suggestion of relative greater importance of NK cells to T cells in controlling virus load and spread, particularly for a virus not previously encountered by the host. None of the 52 community patients with milder symptoms had detectable viral RNA, providing some support for correlation between Ct levels, viral burden and disease severity.

NK cells are a key component of the innate immunity. They represent about 5–20% of circulating peripheral blood lymphocytes and rapidly accumulate in organs during viral infections, tumour growth and inflammation. The role of NK cells in influenza A virus infection is understudied but animal studies have shown that NK cell depletion or mice lacking the full repertoire of the NK cells recognition receptors (NCR-1 deficient mice) exhibited increased morbidity and mortality following influenza A virus infection [20]–[21]. Receptors on NK cells (NKp46) can recognize the viral haemaglutinin, and NK cells can respond to influenza A virus infection by augmenting their cytolytic function [22]–[23], suggesting involvement of these cells during influenza virus infection.

NK cells have both cytotoxic and cytokine-producing functions which are controlled by a complex panel of activating and inhibitory receptors [11]. A set of inhibitory receptors, called killer cell immunoglobulin-like receptors or KIRs have a central role in the regulation of NK cell function. The KIR gene cluster and its ligand HLA class I loci is diverse and there is evidence to suggest that NK cell immune responses are genetically determined to some extent [24]. Certain KIR/HLA compound genotypes resulting in the presence of greater NK activating profile are associated with resistance to Hepatitis C virus and HIV infection, slower HIV disease progression and reproduction success [25]–[28]. In a recent paper, Ahlenstiel G and colleagues showed that these compound genotypic differences resulted in different type I interferon response of NK cells infected with influenza A virus [29]. Thus although it is unclear if the reduction of NK cells in our patients is a primary event increasing the susceptibility of disease or a secondary effect of viral infection, Ahlenstiel's study suggests that the former is possible. On the other hand, influenza A virus can infect NK cells directly [30] and the reduction of NK cells may be a consequence of this or activation-induced cell death.

The loss of NK cells was most striking in Patient 1 who was pregnant. Very little is known as to why pregnant patients are more susceptible to severe disease in influenza virus infection. All six patients in a recent paper on pregnancy and swine-origin H1N1/09 infection were reported to have primary viral pneumonia and ARDS was documented in one of the six deaths [31]. There is evidence that maternal immune responses are diminished during pregnancy. Maternal lymphocytes obtained during the second and third trimester show diminished proliferative responses to soluble antigens and allogeneic lymphocytes [32]–[33] and cell-mediated cytotoxicity, the ability of CD8 T lymphocytes to kill allogeneic or viral-infected cells, was also reduced during pregnancy [34]. Abnormalities in NK cells have received much less attention as a potential cause for immune insufficiency during pregnancy; indeed NK cells are thought to be crucial to the success of pregnancy [35], [36]. Of the two major NK cell subsets in human - the CD56^dim^ (cytotoxic) and the CD56^bright^ (immuneregulatory) populations, CD56^bright^ NK cells are increased in pregnancy. This subset is thought to provide benefit by secreting cytokines, chemokines and angiogenic factors to control trophoblastic invasion of the uterus [35].

The lack of circulating T cells to the conserved Matrix viral protein is not entirely surprising in Patient 1 as it is possible that these cells have homed to the lungs during the acute period. In addition, virus-specific T cells are known to fluctuate during infection [6]. However, we expected some of the remaining activated CD8 effector T cells in Patient 3 (where sampling occurred after the acute illness) to be Matrix-specific T cells since this epitope is identical to the H1N1/09 influenza virus, and particularly as the virus-infected, non-ventilated patient (P4) showed an expansion of these T cells during the acute period. It is also clear but not surprising that some individuals (H5 in Figure 3d) maintain a more sizeable population of antigen-specific T cells than others.

It is noteworthy that the CD4:CD8 T cell ratios were slightly increased in the peripheral blood of the severely ill, ventilated H1N1/09 patients (H1N1/09, ITU in Figure 3a) and even more markedly in the infected non-ITU patients. There is continual debate about the relative contribution of T cells to protection and pathology in influenza virus infection. This finding raises the hypothesis that the ‘cytokine storm’ observed in the serum of some patients could be secondary to circulating CD4 T cells, while CD8 T cells are protective.

In summary, these three sequential severe cases serve as detailed examples of severely ill patients from the new pandemic influenza virus, and from which hypotheses might be generated. From the immunological aspect, the reduction in NK cells raises the interesting possibility of genetic polymorphisms affecting the NK frequency or function; and the relative greater importance of NK cells to T cells in the control of viral load and dissemination during a first encounter with a new virus.
